# Effects of dipeptidyl peptidase-4 inhibition in an animal model of experimental asthma: a matter of dose, route, and time

**DOI:** 10.1002/phy2.95

**Published:** 2013-10-02

**Authors:** Michael Stephan, Hendrik Suhling, Jutta Schade, Mareike Wittlake, Tihana Tasic, Christian Klemann, Reinhard Pabst, Marie-Charlot Jurawitz, Kerstin A Raber, Heinz G Hoymann, Armin Braun, Thomas Glaab, Torsten Hoffmann, Andreas Schmiedl, Stephan von Hörsten

**Affiliations:** 1 Institute of Functional and Applied Anatomy, Hannover Medical SchoolHannover, Germany; 2 Clinic of Psychosomatics and Psychotherapy, Hannover Medical SchoolHannover, Germany; 3 Department of Respiratory Medicine, Hannover Medical SchoolHannover, Germany; 4 Experimental Therapy, Franz-Penzoldt-Center, Friedrich-Alexander-University Erlangen-NürnbergErlangen, Germany; 5 Fraunhofer ITEMHannover, Germany; 6 Probiodrug AGHalle/Saale, Germany

**Keywords:** Airway responsiveness, asthma, CD26, DPP4 inhibitor, Fischer 344 rats

## Abstract

The CD26-associated enzymatic activity of dipeptidyl peptidase-4 (DPP4) as well as the recruitment of CD26^+^ T cells increase under allergic airway inflammation. Furthermore, genetic deficiency of CD26/DPP4 exerts protective effects in experimental asthma. Therefore, CD26/DPP4 might represent a novel therapeutic target in asthma. To study the effects of pharmacological inhibition of DPP4 on allergic airway inflammation the DPP4-inhibitor isoleucine thiazolidide was tested using different doses at different time points (at sensitization, immediately before and simultaneously with the allergen challenge, as well as continuously via drinking water), and different routes (intraperitoneal, oral, and by inhalation). Allergic-like airway inflammation was induced in Fischer 344 rats (Charles River) sensitized against ovalbumin (OVA) using OVA aerosols. Intraperitoneal application of the DPP4 inhibitor showed effects neither at sensitization nor at challenge, whereas a continuous application via drinking water using high doses of the inhibitor led to an aggravation of the histomorphological signs of airway inflammation. In contrast, aerosolization of the DPP4 inhibitor simultaneously with the allergen significantly reduced airway hyperresponsiveness and ameliorated histopathological signs compared to controls. In addition, this treatment resulted in increased mRNA levels of surfactant proteins, suggesting an involvement of DPP4 inhibitors in surfactant metabolism in OVA-challenged rats. Continuous systemic inhibition of DPP4 via the oral route aggravates allergic airway inflammation. In contrast, topical inhibition of DPP4 exerts potential protective effects, and further research in humans is needed.

## Introduction

CD26 is a type II transmembraneous glycoprotein exerting a unique dipeptidyl peptidase-4 (DPP4) enzymatic activity, first described in 1966 (Hopsu-Havu and Glenner 1966). DPP4 abrogates the insulinotropic effect of glucagon-like peptide-1, while DPP4 inhibitors extend it, consecutively promoting the release of insulin. This effect has led to the introduction of DPP4 inhibitors for the clinical treatment of diabetes mellitus type 2 (Mentlein 2005). However, the enzymatic activity of DPP4 is not specific for glucagon-like peptide-1, and DPP4 inhibitors may therefore also interact with other DPP4 substrates, for example, chemokines being ligands at the CCR3 (eotaxin/CCL11, RANTES/CCL5, etc.) or peptides such as neuropeptide Y (NPY). In line with this notion, recent reports provide evidence of an interaction of DPP4 inhibitors with angiotensin converting enzyme (ACE) inhibitors and NPY (Jackson et al. 2008) as well as the chemokine eotaxin (Forssmann et al. 2008).

As CD26/DPP4 is highly expressed in the lungs with its enzymatic activity being not only attributable to epithelial cells, several types of endothelial cells, and fibroblasts (De Meester et al. 1999) but also to submucosal glands of the human bronchus and in human bronchoalveolar lavage (BAL) fluid (van der Velden and Hulsmann 1999), prominent effects of DPP4 inhibitors in this particular organ are not unlikely. Recently, we reported that DPP4 enzymatic activity in the BAL fluid and parenchyma increases after allergen challenge in a rat model of allergic airway inflammation (Schade et al. 2008). These results suggest membrane-bound CD26/DPP4 on lung epithelium playing a pathophysiological role in asthma that might also be affected by DPP4 inhibition.

We reported that the severity of the inflammatory response to ovalbumin (OVA) correlated with the expression of CD26 in different rat strains (Kruschinski et al. 2005), with a largely blunted inflammatory T-cell response in CD26/DPP4-deficient Fischer 344 (F344) rat substrains. Furthermore, when replicating these results, we found that this reduction in eosinophils and T cells was accompanied by both a differential influx of T-regulatory cells and increased levels of regulatory cytokines (Schmiedl et al. 2010).

However, despite this compelling evidence for a role of CD26/DPP4 in asthma, so far the impact of pharmacological inhibition of DPP4 on allergic airway inflammation has not been studied. To investigate whether a pharmacological inhibition of DPP4 might exert protective effects, we analyzed the impact of different doses of the competitive DPP4-inhibitor isoleucine thiazolidide (ile-thia) at various time points using different routes of administration in a rat model of OVA-induced allergic airway inflammation. As this inhibitor ile-thia is unspecific for DPP4 also affecting DPP4 homologues, we furthermore investigated the effects of ile-thia in a rat model of asthma using F344 rats genetically lacking DPP4 expression and DPP4 activity (Karl et al. 2003).

## Methods

### Experimental design and drugs

A comprehensive approach was to screen DPP4 inhibitor–mediated effects systematically at various doses, routes, and time points. Animals were randomly allocated to four experimental setups (Table 1). In the first setting, four doses (0, 0.1, 1.0, and 10 mg/kg/bw or 0, 1, 5, and 10% as aerosols) were investigated following a single OVA challenge (*n* = 5/group) in rats sensitized against OVA (see below). Subsequently, the dose of inhibitor being most effective for the respective treatment regime was reinvestigated in more animals (*n* = 10/group). Additionally, this dose was also applied in a model of repeated allergen challenge (daily challenge over 3 days; *n* = 10/group), in order to investigate whether DPP4 inhibition exerts any long-lasting immunomodulatory effect (especially, histomorphologic changes) upon exposure to the allergen.

**Table 1:** Experimental design for screening effects of dipeptidyl peptidase-4 (DPP4) inhibition on allergic airway inflammation

	I	II	III	IV
Dose	0, 0.1, 1, **10** (mg/kg)	0, 0.1, 1, 10 (mg/kg)	0, 0.1, 1, 10 (mg/kg)	0, 1, **5**, 10%
Route	Oral via drinking water	i.p. injection	i.p. injection	Topical as an aerosol
Time	Continuously over 3 weeks	At the time point of sensitization	3 h before challenge	Simultaneously with the OVA challenge

All rats were randomly allocated to four experimental conditions. Initially, all four doses administered were investigated following a single OVA challenge (*n = 5/group*). Subsequently, the most effective dose for each treatment regime (dose highlighted in bold) was reinvestigated following a single OVA challenge (*n = 5/group*) as well as after three consecutive allergen challenges (each *n = 10/group*).

The reversible, competitive DPP4-inhibitor ile-thia was provided by Probiodrug AG (Halle/Saale, Germany) (Pederson et al. 1998) and all experiments were performed using a single batch of this compound. Based on the average drinking volume (about 25 mL/day/rat), the drug concentration in the drinking water, and the average body weight (bw) of the rats (250 ± 20 g), the following concentrations per rat and 24-h fluid uptake were used: 0.1, 1.0, or 10.0 mg. Inhalation of the inhibitor was performed simultaneously with OVA challenge. For invasive measurement of airway hyperresponsiveness (AHR), the amount of the inhibitor was estimated with respect to tidal volume, particle size, as well as time of inhalation (Hoymann 2007, 2012). For each of these treatments, drug plasma levels were determined by Probiodrug AG, being sufficient to inhibit plasma DPP4 enzymatic activity at the 1.0 and 10 mg/kg/bw doses as well as the 5% and 10% aerosol dosages.

### Animals

Throughout the experiments, 12-week-old male F344 rats (Charles River, Sulzfeld, Germany) or male CD26/DPP4-deficient F344 rats (F344/Crl(Wiga)SvH-*Dpp4*^*m*^) (only as a comparison group) (Karl et al. 2003) were used. Rats were housed under standard conditions and were microbiologically monitored according to FELASA recommendations (Rehbinder et al. 2000). The government of Lower Saxony, Oldenburg, Germany, approved all research and animal care procedures.

### Allergic airway inflammation and functional airway parameters

For the induction of allergic airway inflammation, rats were sensitized 14 and 7 days before challenge, as previously described (Skripuletz et al. 2007). In brief, all rats were sensitized against OVA (1 mg Grade V; Sigma, Taufkirchen, Germany; batch: 025K7001) and were challenged by aerosolizing 5% of an OVA solution (2 mL). Simultaneously, the early airway response (EAR) to OVA was assessed by recording the midexpiratory flow (EF50) by head-out body plethysmography, as previously described (Glaab et al. 2002). AHR in response to acetylcholine as indicated by enhanced pulmonary resistance and a decrease in EF50 was invasively determined in allergic rats 24 h after the first aerosolized OVA challenge (Glaab et al. 2002; Hoymann 2007). For measurement, rats were anesthetized with isofluran and orally intubated as previously described (Hoymann 2012).

### Organ sampling, processing, and analyses

Rats were dissected and samples from blood, BAL fluid, and lungs were obtained for further analyses 22 h after the last challenge, as previously described (Skripuletz et al. 2007). All three compartments were investigated by fluorescence activated cell sorting (FACS) analysis using commercially available antibodies (all from Serotec, Duesseldorf, Germany) (Pabst et al. 2003). Cells obtained from the BAL fluid and blood were further differentiated by cytospins. Antigen-specific IgE levels in the blood were investigated by enzyme-linked immunosorbant assay (ELISA) (Rehbinder et al. 2000). Whole left lungs were processed for immunohistological stainings, and eosinophilic infiltration of alveoli, septa, and perivascular space was calculated per mm^2^ by random sampling (Skripuletz et al. 2007). Lung parenchyma of the inferior lobe of each right lung was bisected and either shock frozen for polymerase chain-reaction (PCR) analyses of IL-4 (fw:TCCTTACGGCAACAAGGAAC; rev:GTGAGTTCAGACCGCTGACA), IFN-γ (fw:GCCCTCTCTGGCTGTTACTG; rev:CTGATGGCCTGGTTGTCTTT), the surfactant proteins A (fw:CTAAGTGCTGCCCTCTGACC; rev: AGGAGCCATACATGCCAAAC), B (fw:CTGTGCCAAGAGTGTGAGGA; rev:CAAGCAGCTTCAAGGGTAGG), C (fw:CAGCTCCAGGAACCTACTGC; rev:CTCTCCACACAAGGTGCTCA), and D (fw:ATGGCCAAAGTGTTGGAGAC; rev:CGTGCCCACATCTGTCATAC), or homogenized for separation of cells for ex vivo culture as previously described (Kruschinski et al. 2005). Portions of 10^6^ cells were isolated, stimulated with 1-μg Con-A per well, and cultured for 72 h. Subsequently, IFN-γ, IL-4, and IL-10 were measured in the supernatant using commercially available ELISA kits (all from BD Pharmingen, Heidelberg, Germany). Plasma levels of the inhibitor were measured using liquid chromatography–mass spectrometry.

### Statistics

Statistical analysis was performed using analysis of variance (ANOVA) with the factor “treatment.” *P*-values <0.05 were considered significant and are indicated by asterisks. All data are given as arithmetic mean ± SEM.

## Results

### Continuous oral inhibition of DPP4 aggravates the inflammatory response to OVA

Having previously reported a blunted inflammatory response to OVA in CD26/DPP4-deficient F344 rats using a model of allergic lung inflammation (Schmiedl et al. 2010), we tried to mimic this genetically induced status of CD26/DPP4 deficiency using a pharmacological approach by a continuous application of the DPP4-inhibitor ile-thia via drinking water, starting 3 days before the first sensitization. Application of the DPP4 inhibitor via the drinking water did not lead to a significant alteration of bw or of water consumption (no test aversion). In initial dose-finding experiments, doses of the DPP4 inhibitor of 0.1 and 1 mg/kg/bw/24 h turned out to have no significant effects. Therefore, a dose of 10 mg/kg/bw/24 h via drinking water was selected for consecutive experiments resulting in a mean plasma level of 4.2 ng/mL (interquartile range [IQR] 2; 9) at time point of organ harvesting.

**Figure 1:**
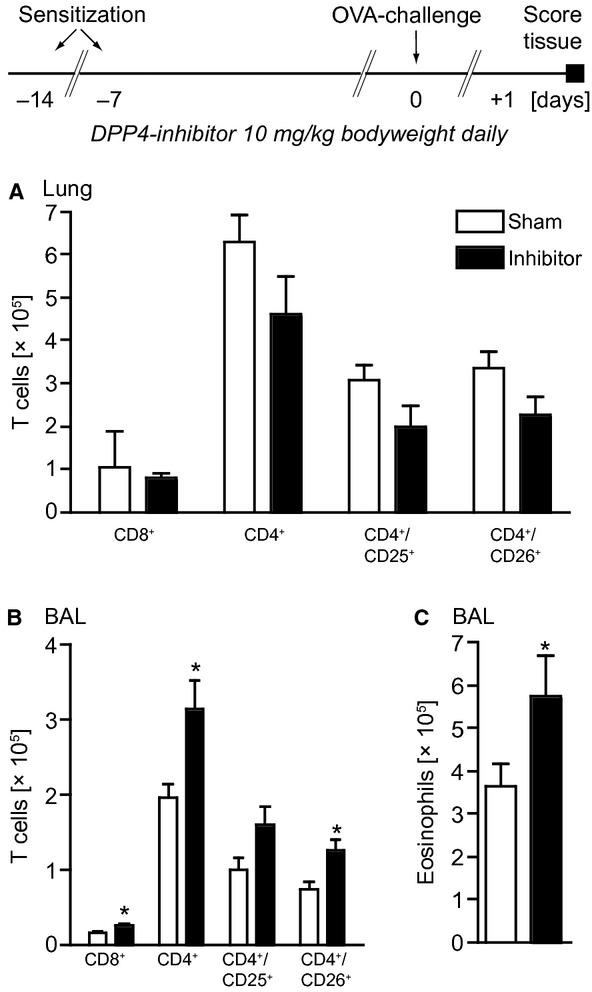
Continuous dipeptidyl peptidase-4 (DPP4)-inhibition aggravates allergic airway inflammation following single allergen challenge. Continuous inhibition via drinking water started 3 days before the first sensitization. Twenty-two h after a single ovalbumin (OVA) challenge, there were no significant effects for T cells in the lung parenchyma (A), but a significant increase in the amount of CD4^+^ and CD8^+^ T cell subpopulations (B) as well as in eosinophils (C) was found in the bronchoalveolar lavage (BAL) fluid.

A histological screening revealed no significant differences between sham controls and the treatment group following a single OVA challenge, although there was a trend to a higher influx of eosinophil granulocytes after inhibitor treatment. In the lung tissue, there were no differences concerning the recruitment of T cells, as obtained by flow cytometry (Fig. 1A). Ex vivo studies investigating the supernatant of cells separated from lungs as well as PCR analyses on lung homogenates showed no significant alterations of IFN-γ, IL-10, and IL-4 protein or mRNA levels (data not shown). However, in the BAL fluid, the absolute number of cells significantly increased under treatment conditions (29.9 ± 4.4 × 10^6^) compared to sham controls (16.0 ± 1.8 × 10^6^). This was accompanied by a significant increase in CD8+ and CD4+ T cells (Fig. 1B) and eosinophils (Fig. 1C).

In the clinical situation, several parameters in the peripheral blood are indicative of an allergic state of the individual, one of these being the expression of CD26 on CD4+ T cells (Pabst et al. 2003). However, in this study no significant differences for the numbers of CD26+ T cells (sham group: 6.9 ± 0.4 × 10^5^, inhibitor group: 7.1 ± 0.7 × 10^5^) or CD4+/CD26+ T cells (sham group: 4.8 ± 0.2 × 10^5^, inhibitor group: 4.8 ± 0.4 × 10^5^) were found following inhibitor treatment in the blood. The OVA-specific IgE plasma levels also remained unaffected (sham group: 0.9 ± 0.1, inhibitor group: 1.0 ± 0.1; arbitrary units).

Investigating functional aspects of the pharmacological inhibition, there were neither significant differences in the EAR nor any persistent effects for the full range of doses in the AHR against acetylcholine.

**Figure 2:**
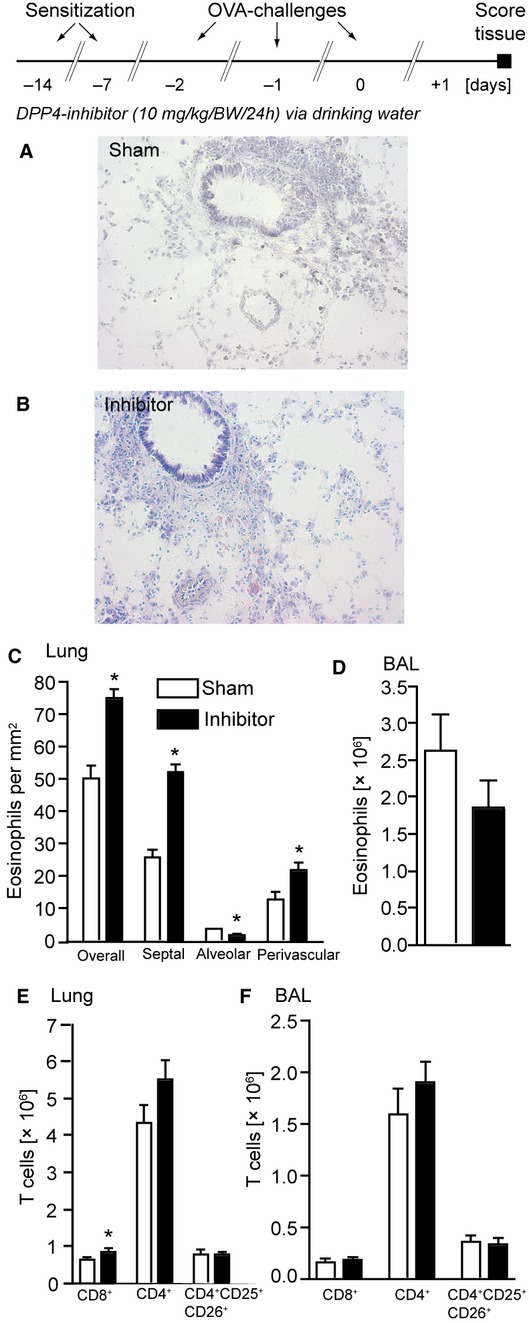
Continuous dipeptidyl peptidase-4 (DPP4) inhibition increases eosinophil recruitment to the lungs after repeated allergen challenges. Histopathological signs of airway inflammation 22 h after the last of three consecutive ovalbumin (OVA) challenges are similar in both groups (Giemsa staining). (A, B) DPP4 inhibition led to pronounced eosinophilia in the lungs (C), whereas the recruitment to the bronchoalveolar lavage (BAL) (D) remained unaffected. However, the amount of CD4^+^ T cells in the lungs (E) and the bronchoalveolar lavage (BAL) (F) was not significantly altered.

To study whether repeated OVA challenges lead to comparable results, the impact of a continuous DPP4 inhibition on histopathological signs was investigated after daily allergen challenges repeated three times. A more pronounced eosinophilic inflammation was found in the lung septa and the perivascular space (Fig. 2A–C) as well as an increased number of T cells (Fig. 2E). However, the penetration of these cells into the airways, as displayed by the BAL fluid, was not altered by oral inhibitor treatment (Fig. 2D, F).

### No effects of intraperitoneal inhibition of CD26/DPP4 either at sensitization or three hours before challenge

To investigate whether the observed changes under a continuous inhibition of DPP4 were due to either inhibitor-mediated effects at the time point of sensitization or at challenge, the impact of different inhibition strategies was investigated using several doses of ile-thia. However, the concentrations of the DPP4 inhibitor chosen (0.1, 1.0, and 10.0 mg/kg bw i.p.) led to no significant differences for histological scores or functional parameters in comparison to sham treatment either at the time point of sensitization or 3 h before challenge.

### Aerosolized DPP4 inhibitor ameliorates the allergic inflammation after a single challenge

To investigate whether a topical application of the DPP4 inhibitor alters the asthmatic reaction to a simultaneously nebulized antigen, concentrations of 1%, 5%, as well as 10% of the DPP4 inhibitor were tested. None had any effects on airway responsiveness or leukocyte composition in the BAL fluid, when aerosolized alone without an OVA challenge.

We decided to investigate the effects of a 5% solution of the DPP4 inhibitor as the other concentrations used showed slightly minor effects on allergic airway inflammation when aerosolized simultaneously with the antigen. The mean plasma inhibitor concentration was 14.5 ng/mL (IQR 3; 59). The calculated average deposit was 75 μg inhibitor in each rat.

**Figure 3:**
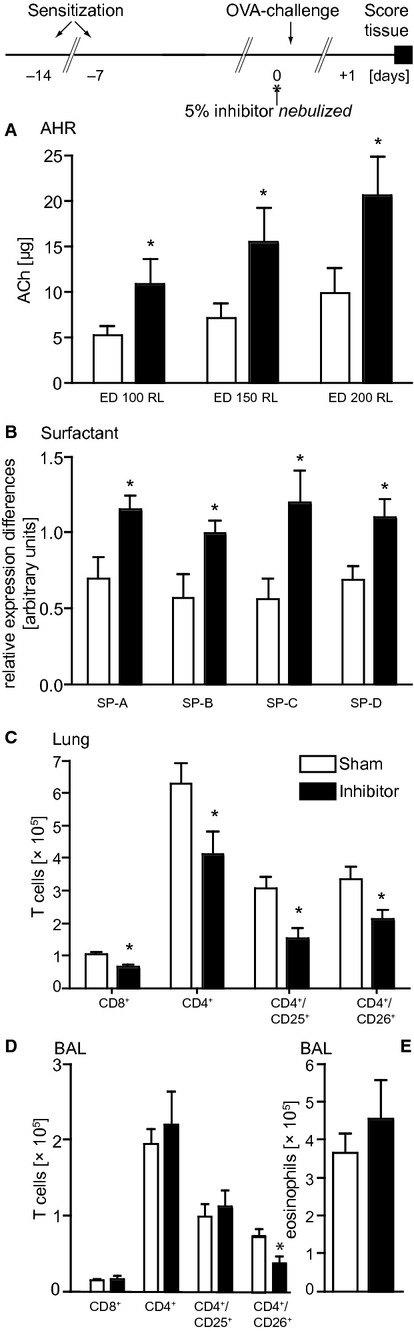
Topical inhibition of dipeptidyl peptidase-4 (DPP4) ameliorates signs of allergic airway inflammation following single allergen challenge. There was a significant amelioration of airway hyperresponsiveness (AHR) after treatment with aerosolized inhibitor (A). On abscissa, the effective inhalational dose (ED) is given. This dose was defined as the cumulative dose of required ACh (given in μg on ordinate) to increase airway resistance by 100, 150, or 200% compared to baseline. This was accompanied by increased mRNA levels of all surfactant proteins (B). Furthermore, the number of T cells decreased significantly indicating reduced signs of inflammation in the lungs (C), whereas no effects were found in the bronchoalveolar lavage (BAL) fluid except for a reduction in CD4^+^T cells-expressing CD26 (D, E).

While a local inhibition of DPP4 via the airways failed to alter the EAR after a single challenge, it significantly increased the amount of cholinergic AHR compared to untreated controls (Fig. 3A). In CD26/DPP4-deficient F344 rats there was an upregulation of surfactant found in the BAL (Schmiedl et al. 2010). Here, we also found increased levels of mRNAs coding for each of the four surfactant proteins (Fig. 3B). Additionally, the number of T cells recruited to the lungs decreased under this treatment (Fig. 3C), indicating an attenuated inflammatory response. However, in the BAL fluid, there was only a trend to decreased numbers of the overall amount of T cells with the exception of T cells–expressing CD26+, which were significantly reduced (Fig. 3D). Interestingly, the mean expression of CD26 on T cells increased significantly following DPP4-inhibition (138 ± 17 arbitrary units/mean fluorescence intensity) compared to controls (74 ± 3 arbitrary units; *P* = 0.001), indicating an increase in the mean expression of this activation markers on each cell, whereas opposite effects were observed for CD25 (inhibitor group: 169 ± 10, sham group: 239 ± 14, arbitrary units; *P* = 0.0009). The numbers of eosinophils in the BAL fluid remained unaffected (Fig. 3E). Again, neither ex vivo ELISAs nor PCR analyses revealed significantly altered levels of cytokines in the lungs (data not shown). In the peripheral blood, a trend toward reduced levels of CD25+ and CD26+ T cells as well as OVA-specific IgE levels was found following inhibition of DPP4.

**Figure 4:**
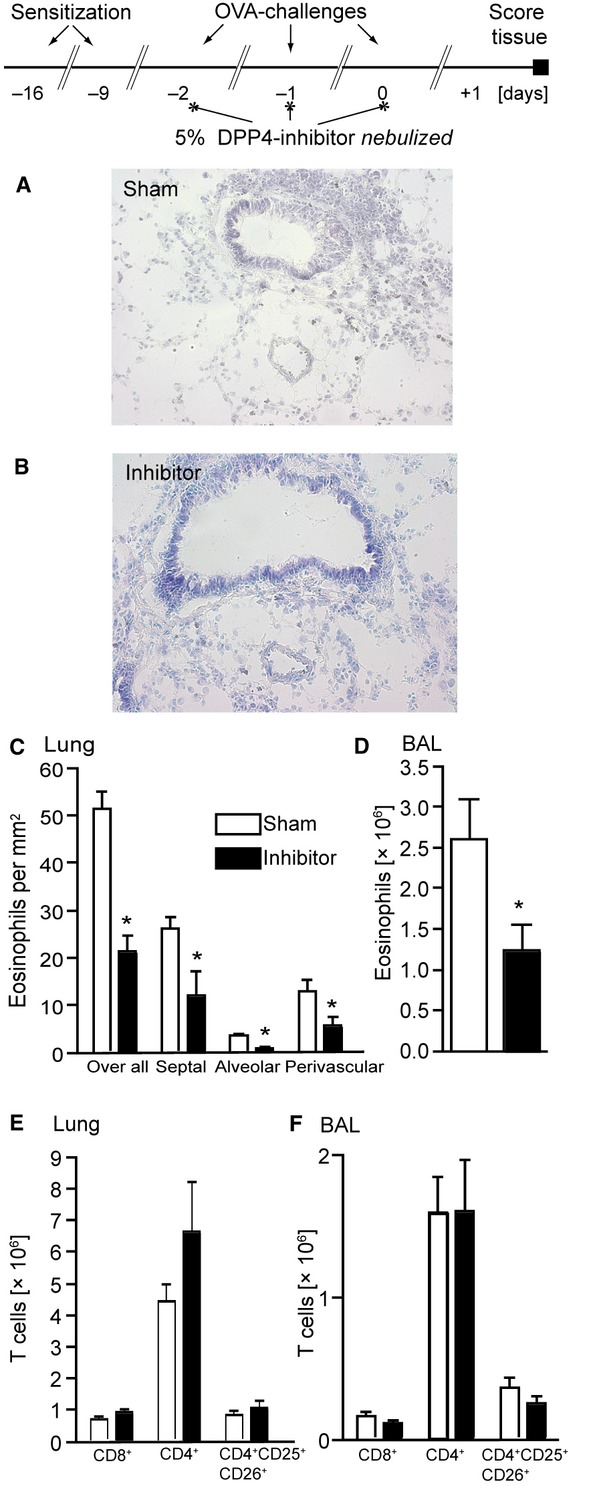
Aerosolization of dipeptidyl peptidase-4 (DPP4) inhibitor reduces eosinophilic inflammation after repeated allergen challenges. Representative micrographs (Giemsa staining) illustrate inflammation in lung parenchyma of sham controls (A) that was reduced in inhibitor-treated rats (B). Quantification of eosinophils shows a reduced eosinophilia in all lung compartments investigated (C) as well as in the bronchoalveolar lavage (BAL) (D), whereas the amount of T cells in the lungs and the BAL was unaffected (E, F).

To investigate whether topical DPP4-inhibitor treatment exerts any long-lasting immunomodulatory effects additionally upon reexposure to the allergen, histomorphological signs of inflammation were studied following three consecutive OVA challenges. Here, again we found a significant amelioration of the inflammatory response due to reduced signs of inflammation and especially decreased levels of eosinophils recruited to all compartments of the lungs measured (Fig. 4A–D). Although the T-cell levels in the lungs as well as in the BAL fluid remained unaffected (Fig. 4E, F), there was a significant reduction in eosinophils in the BAL fluid of almost 50% (Fig. 4D), indicating beneficial effects of topical DPP4 inhibition in a model of repeated allergen challenges.

### Pharmacological inhibition of DPP4 exerts no effects on allergic lung inflammation in DPP4-deficient rats

We tested the effects of the unspecific inhibitor ile-thia in DPP4-deficient rats. Beneficial effects in rats genetically lacking DPP4 expression as well as DPP4 activity would have pointed to a mechanism involving other enzymes, for example, DPP8 or DPP9 (Schade et al. 2008). However, no significant effects were using DPP4-deficient rats, either concerning the number and the composition of cells in the BAL fluid or the airway responsiveness (data not shown). The airway inflammation was at significantly lower levels in untreated F344-deficient rats compared to untreated wild-types effects, confirming previous results (Schade et al. 2009; Schmiedl et al. 2010).

## Discussion

This is the first study dealing with DPP4 inhibitor–mediated effects on allergic responses of the airways. We were able to show a distinct route-, time-, and dose-dependent modulation. Until now, it has been hypothesized that CD26/DPP4 plays a crucial role in the onset and course of allergic inflammations (Ohnuma et al. 2005), but to our knowledge no experimental studies trying to modulate asthma have been published. The experiments conducted in this study demonstrate that in F334 rats continuous oral treatment using DPP4 inhibitors leads to an aggravation of the inflammatory response, whereas nebulizing the inhibitor simultaneously with the allergen might attenuate airway responsiveness.

CD26 has been described as a T-memory marker and thus as being involved in IL-2 production, at least in humans (Morimoto and Schlossman 1998). Hypothesizing that a CD26^bright^ “late-memory” T-cell subpopulation plays an important role in the sensitization to an allergen, a systemic inhibition of CD26/DPP4 at the time point of sensitization should have implications for the course of asthma. In this part of the study, surprisingly, we were not able to alter the outcome of the disease. One explanation might be the substantial differences in the role of CD26 in T-cell activation between humans and rodents, as already reported for mice (Cordero et al. 2007). In mice, CD26 is not directly involved in the conversion of T cells from the naïve to the memory phenotype. In rats, the impact of CD26 on T-cell conversion has not been investigated, but the fact that pharmacological inhibition at the time point of sensitization had no effects indicates that the differentiation of T cells to a memory phenotype is unlikely to be altered by short-term DPP4-inhibitor treatment in vivo in this species. Supporting this, experiments have revealed a lack of differences in the composition of T-cell subpopulations and especially in memory T cells in a rat model of genetic DPP4 deficiency at the age of 3 months (Klemann et al. 2009).

In contrast to the beneficial effects of a genetically induced CD26/DPP4 deficiency in a rat model of experimental asthma (Kruschinski et al. 2005), an aggravation of the inflammatory response was found under a continuous oral pharmacological inhibition of DPP4. Inflammatory cells increased in both a more acute, T cell–driven model as well as in a model of repeated allergen challenges, showing a pronounced allergic-like, eosinophil-driven inflammation. Although functional outcomes such as EAR and AHR remained unaffected, these findings point to immunomodulator effects and imply clinical relevance, as DPP4 inhibitors have been introduced as novel antidiabetics (Gautier et al. 2005), with undesired effects possibly being underestimated (Nathan 2007). However, although our data indicate potential adverse effects of a continuous DPP4 inhibition, it should clearly be stated that the immunomodulatory effects observed occurred only using high doses of the DPP4 inhibitor.

In contrast to the aggravation induced by continuous DPP4 inhibition, our finding that topical DPP4 inhibition induces a significant amelioration of clinical as well as histomorphological signs of allergic airway inflammation implicates potential therapeutic relevance. As there is no expression of DPP4, but only DPP8 and DPP9 in the great and middle air-conducting bronchi (Schade et al. 2008), we accordingly found no amelioration of the EAR. However, a significant improvement of cholinergic AHR by topical DPP4 inhibition was observed. As demonstrated in Figure 3, almost twice as much acetylcholin (ACh) was needed to induce an equivalent airway obstruction in rats that were treated with inhibitor. This might be at least partly due to increased surfactant protein levels, which are known to contribute to the reduction in surface tension (Mallory 2001; Erpenbeck et al. 2006) and thereby ameliorating AHR (Takeda et al. 2003).

Analogous to the effects previously shown in a genetic model of CD26/DPP4 deficiency, a pharmacological CD26/DPP4 inhibition increases surfactant proteins under allergic-like airway conditions (Schmiedl et al. 2010). The attenuated inflammatory response might contribute to the observed amelioration of clinical signs (Hohlfeld 2002; Hallman 2004).

The observed differences depending on route of inhibitor administration might be explained by:

1. The increased local inhibition of bronchial epithelial bound DPP4 resulting in reduced local chemotaxis (Hallman 2004). Nevertheless, the administration by inhalation and the higher concentration of inhibitor in the airway must be the key to different effects. As uptake of DPP4 inhibitor is possible by intestinal peptide transporter 1 (PEPT1) and pulmonary PEPT2 peptide transporter and levels of the inhibitor were detectable in the peripheral blood, the higher concentration in the lung must be the crucial factor. Due to dilution effects in the BAL fluid, a measurement of ile-thia was not possible.
2. DPP4 inhibition might directly influence CD26 being expressed on endothelial cells, thereby affecting adhesion and transmigration of inflammatory cells.
3. A significantly increased production of mRNAs was found for all four surfactant proteins following DPP4 inhibition, indicating an altered activation pattern of alveolar epithelial cells, which is worth further investigation.
4. It has been shown that the T cell–driven airway inflammation in rats is mainly based on CD4+/CD25+/CD26+ T cells, which were distinct from regulatory T cells (Skripuletz et al. 2007). It has become apparent that CD26 on T cells interacts with calveolin-1 of antigen-presenting cells, resulting in an upregulation of CD80 and CD86 on these cells and thereby enhancing the bond of the immunological synapse (Ohnuma et al. 2004). Furthermore, calveolin-1 is also a costimulatory ligand for CD26 in T cells, with a ligation-inducing T-cell activation and proliferation (Ohnuma et al. 2007). These effects demonstrate CD26 to be an important molecule in T-cell stimulation.

In conclusion, a topical inhibition of DPP4 has beneficial effects on allergic symptoms, whereas a continuous inhibition can aggravate the course of an allergen-induced inflammatory response. These effects might be due to an inhibition of DPP4 on T cells or endothelial DPP4, as the observed effects are not evident in DPP4-deficient F344 rats. This might pinpoint to DPP4 instead of DPP8 or DPP9 being the pharmacological target. However, it has to be taken into account that DPP4-deficient rats already show lower responses to OVA (Schade et al. 2009; Schmiedl et al. 2010). As the inflammatory response of the airways is already blunted in DPP4-deficient rats, the observed effect of no further amelioration by DPP4 inhibition is of limited explanatory power.

As the model of induced airway obstruction in rats does not reflect typical human asthma, further studies are needed to identify CD26 inhibition and involvement in humans. A limitation of the study was that in repeatedly allergen-challenged rats measurements of cytokine and AHR were not undertaken.

While CD26/DPP4 seems to be an interesting possible novel therapeutic target when topically inhibited, undesirable effects have to be apprehended and the continuous use of oral DPP4 inhibitors should be carefully monitored in comorbid patients suffering from diabetes mellitus type 2 as well as from asthma or other allergic diseases.
